# Patterns and drivers of daily bed-level dynamics on two tidal flats with contrasting wave exposure

**DOI:** 10.1038/s41598-017-07515-y

**Published:** 2017-08-02

**Authors:** Zhan Hu, Peng Yao, Daphne van der Wal, Tjeerd J. Bouma

**Affiliations:** 1 Institute of Estuarine and Coastal Research, School of Marine Science, Sun Yat-sen University, Guangzhou, 510275 China; 2 Guangdong Provincial Key Laboratory of Marine Resources and Coastal Engineering, Guangzhou, 510275 China; 3 State-province Joint Engineering Laboratory of Estuarine Hydraulic Technology, Guangzhou, 510275 China; 4 Royal Netherlands Institute for Sea Research (NIOZ), P.O. Box 140, 4400 AC Yerseke, Netherlands

## Abstract

Short-term bed-level dynamics has been identified as one of the main factors affecting biota establishment or retreat on tidal flats. However, due to a lack of proper instruments and intensive labour involved, the pattern and drivers of daily bed-level dynamics are largely unexplored in a spatiotemporal context. In this study, 12 newly-developed automatic bed-level sensors were deployed for nearly 15 months on two tidal flats with contrasting wave exposure, proving an unique dataset of daily bed-level changes and hydrodynamic forcing. By analysing the data, we show that (*1*) a general steepening trend exists on both tidal flats, even with contrasting wave exposure and different bed sediment grain size; (*2*) daily morphodynamics level increases towards the sea; (*3*) tidal forcing sets the general morphological evolution pattern at both sites; (*4*) wave forcing induces short-term bed-level fluctuations at the wave-exposed site, but similar effect is not seen at the sheltered site with smaller waves; (*5*) storms provoke aggravated erosion, but the impact is conditioned by tidal levels. This study provides insights in the pattern and drivers of daily intertidal bed-level dynamics, thereby setting a template for future high-resolution field monitoring programmes and inviting in-depth morphodynamic modelling for improved understanding and predictive capability.

## Introduction

Tidal flats are highly important ecosystems, given their ecosystem services including providing habitats for unique plants and benthic invertebrates^1–3^, contributing to coastal defence^4–6^, as well as hosting migratory birds^7–9^. Hence, many existing field and modelling studies seek to understand medium to long-term tidal flat morphodynamics. The fate of tidal flats and associated vegetation ecosystems under the threat of sea level rise has been discussed in depth^10–14^. The roles of wave and tidal forcing in long-term tidal flat evolution were also systematically explained^15–19^. In contrast, only very few studies have investigated bed-level variations on daily or shorter scales^20–22^, even though these short-term (daily) bed-level fluctuation may be the key factors controlling the biogeomorphic and ecological functioning of these tidal flats.

Recent studies have shown that such short-term (daily) bed-level fluctuation on tidal flats form a major disturbance to the newly settled benthic invertebrates and vegetation seedlings^23–29^. When the bed-level fluctuation exceeds a certain threshold, the newly-settled seedlings or benthos can be buried or dislodged from the bed, causing establishment failure. Furthermore, the short-term bed-level dynamics can induce the onset of cliff erosion in saltmarsh systems, forming one of the critical tipping points for cyclic saltmarsh dynamics^29,30^. As these short-term (daily) bed-level fluctuations are thus key to controlling the ecological development and thereby biogeomorphic functioning of these tidal flats, we elucidate the processes controlling these crucial but understudied dynamics.

The few existing studies focussed on short-term (daily) bed-level fluctuation on tidal flats have analysed local morphological response to waves, tidal currents and their combined effects in details, but they typically had limited temporal (e.g., 2–5 tidal cycles) or spatial resolution (e.g., 1–2 stations)^20–22,31^, due to a lack of suitable method to obtain high resolution data while avoiding excessive labour^32,33^. Hence, the spatiotemporal pattern of daily bed-level dynamics remains largely unexplored. Only in rare occasions, high-resolution bed-level data was obtained by long-term surveys^32,34^, showing large short-term bed-level variability compared to net annual bed-level change. Strong erosion during storm events was also highlighted^32,34^. However, in these studies, bed shear stress (BSS) induced by wave and tidal current as the chief hydrodynamic forcing was not analysed in concert with the daily bed-level changes. Thus, the relation between hydrodynamic forcing and the spatiotemporal pattern of short-term bed-level dynamics remains unknown.

**Figure 1:**
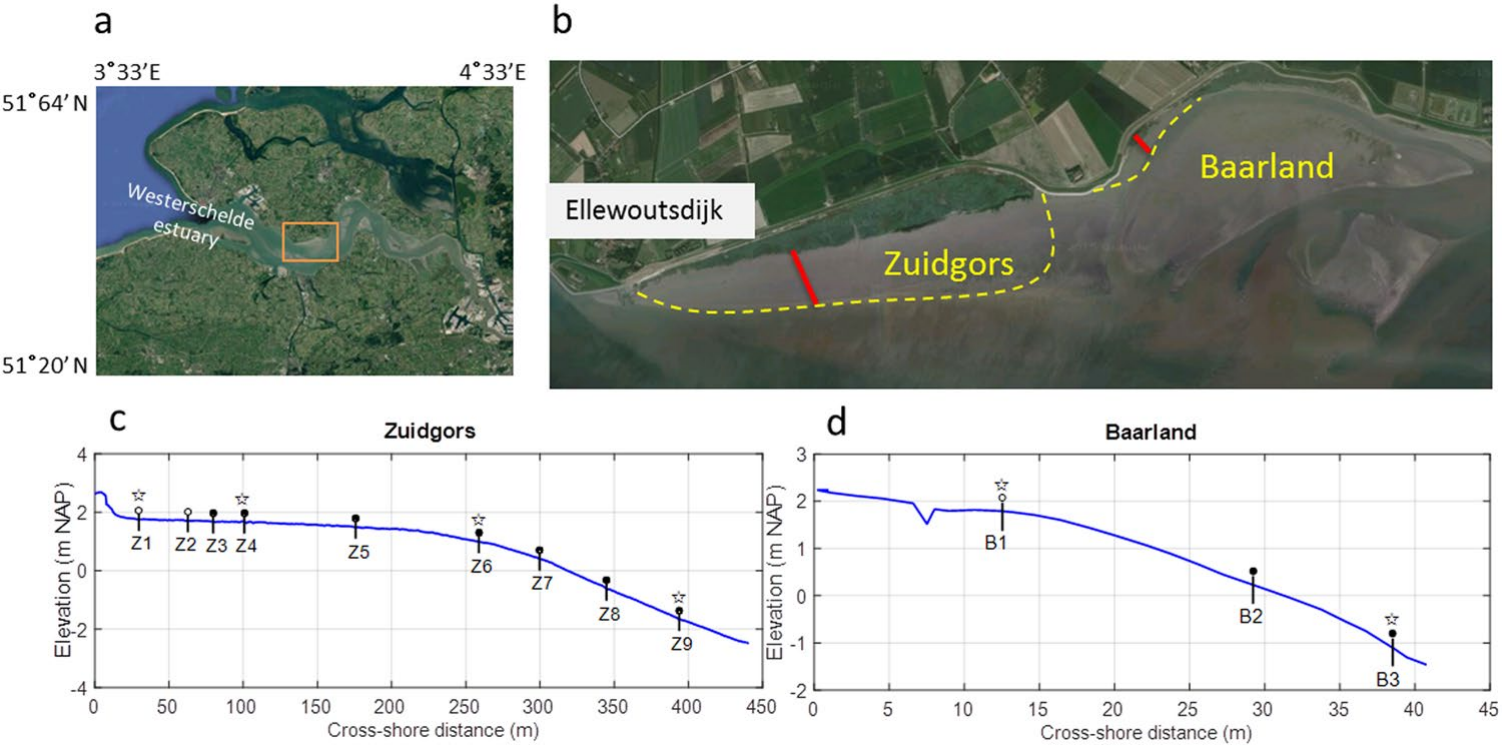
Study site at the northern bank of the Westerschelde estuary, south-west of the Netherlands (plane **a**). Two tidal flats (Zuidgors and Baarland) near the town Ellewoutsdijk were monitored (plane **b**). The seaward boarder of these two tidal flats are indicated by yellow dash line. The Zuidgors site is directly exposed to waves, whereas the Baarland site is sheltered from waves by a seaward shoal. 9 stations (Z1-Z9) were deployed at the Zuidgors site, and 3 stations (B1-B3) were deployed at the Baarland site (plane **c** and **d**). Elevation is relative to NAP (Normal Amsterdam Peil). An SED-sensor is deployed at each station. Pressure sensors for wave measurement and ADCP for velocity measurement are indicated by filled circles and open stars, respectively. Stations with open cycles do not have pressure sensors installed. Images in panel a and b are from Map data ©2017 Google.

In the current study, we aim to (*1*) reveal the general pattern of daily bed-level dynamics at two sites with contrasting wave exposure as the wave-exposed site is expected to show greater bed-level fluctuations than the wave-sheltered site; (*2*) understand the respective roles of tides and waves in driving both the daily bed-level dynamics under storm and calm weather, as well as controlling the relatively long-term (15 months) dynamics; (*3*) provide a template for future field studies on short-term intertidal bed-level dynamics with automatic high-resolution monitoring networks. Two tidal flats with high and low wave exposure, i.e. Zuidgors and Baarland in the Westerschelde Estuary, the Netherlands, were chosen as study sites (Fig. 1). The hydrodynamic forces at these two sites may not be totally contrasting, but the relative strength of wave or tidal current forcing can be different. We hypothesize that at the site with high wave exposure the daily bed-level dynamics is significantly related to the daily wave forcing, whereas at the site with low wave exposure the daily bed-level dynamics is insignificantly related to its daily wave forcing. Newly-developed SED-sensors (Surface Elevation Dynamics sensors) were distributed over the two sites to obtain high-resolution data of bed-level dynamics^33^. On the Zuidgors site, we deployed 9 stations, i.e. Z1 to Z9 from the marsh edge towards to the seaward channel. On the Baarland site, the cross-shore distance is much shorter compared to that of the Zuidgors site. Thus, we deployed only 3 stations, i.e. B1 to B3 from the upper tidal flat to the seaward channel. From n = 3 onwards, one can calculate meaningful correlations. BSS induced by wave and tidal current was quantified by numerical models and subsequently analysed in concert with the bed-level dynamics. The impact of tidal and wave forcing on bed-level dynamics was investigated primarily via correlation analysis. Storm impacts during neap and spring tide were demonstrated systematically.

## Results

### Different hydrodynamic forcing at two sites

**Figure 2:**
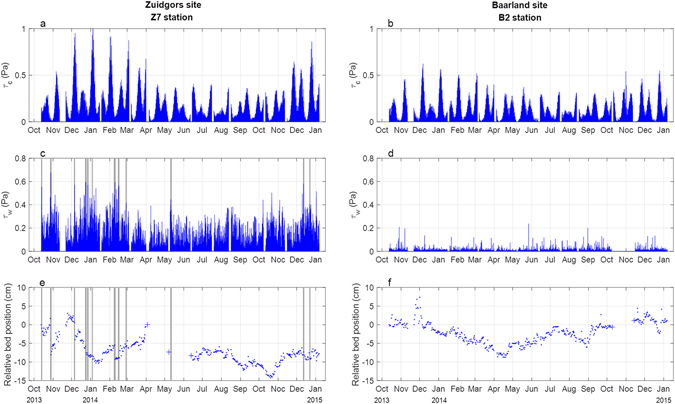
Tidal current induced BSS (*τ*_*c*_), wave induced BSS (*τ*_*w*_) and daily relative bed positions at the exposed Z7 and sheltered B2 station. *τ*_*c*_ and *τ*_*w*_ are obtained by hydrodynamic models. The daily bed-level position data is obtained by newly-developed SED-sensors. The bed-level at the start of the measurements is at ‘0’. The positive and negative values indicate deposition and erosion, respectively. When SED-sensor failure leads to no daily bed-level data, the monthly-obtained differential GPS data is used as substitute (the ‘+’ sign in panel e and f). The shaded stripes indicate stormy periods, i.e. when the incident significant wave height at the wave-exposed site exceeds 0.5 m (panel c and e).

The two monitored tidal flats have similar *τ*_*c*_ (i.e. BSS induced by tidal current) but contrasting *τ*_*w*_ (i.e. BSS induced by wave), which may induce different daily bed dynamics (Fig. 2). Comparing the hydrodynamic characteristics for both field sites at points with a comparable elevation (0.3–0.45 NAP for Z7 and 0.2–0.3 NAP for B2) shows that at both stations, the peak tidal forcing varies seasonally: i.e. stronger *τ*_*c*_ during Dec to Mar versus weaker *τ*_*c*_ during May to Nov (Fig. 2a and b). Within each month, *τ*_*c*_ varies in spring-neap cycles. *τ*_*c*_ at the Baarland site is smaller compared to that at the Zuidgors site, which may be attributed to the inner location of this site (Fig. 1). Wave forcing (*τ*_*w*_) at both sites is considerably different, with the exposed Zuidgors site having a much stronger *τ*_*w*_ compared to the sheltered Baarland site (Fig. 2c and d). In fact, the *τ*_*w*_ at the Baarland site rarely exceeds 0.1 Pa, which is almost negligible.

### Spatial pattern of bed-level dynamics and BSS distribution

The mean bed level position in Nov-2013 to Jan-2014 is at −2.89 cm, and it was lower to −8.21 cm in Nov-2014 to Jan-2015 (Fig. 2e). Thus, the Z7 station is in net erosion over a one year time frame. At the B2 station, the bed-level experiences erosion in Dec–Apr and recovery in May–Dec (Fig. 2f). From Dec to Apr, the peak *τ*_*c*_ at the Z7 station is almost twice as high as that at the B2 station, but the observed net erosion at the Z7 station is only 2.15 cm, much smaller compared to 11.1 cm erosion at the B2 station. Furthermore, large short-term bed-level variabilities can be observed at both stations. Notably, storm events lead to short *τ*_*w*_ peaks and sudden erosion at the exposed Z7 station (Fig. 2e). For the complete bed-level data sets at all stations, please see the Supplementary Information.

**Figure 3:**
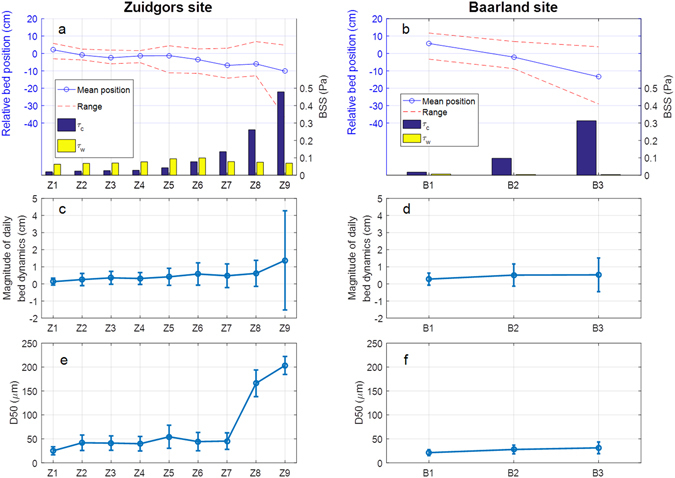
Spatial distribution of relative bed-level positions, *τ*_*c*_ and *τ*_*w*_ (panel a and b), magnitude of daily bed-level dynamics (panel c and d), and median sediment grain diameter (D50) (panel e and f) at the exposed Zuidgors and sheltered Baarland site. The relative bed-level positions are comparing to the starting position at ‘0’, which indicates overall spatial morphodynamic trends. The bed-level at the start of the measurements is at ‘0’. The positive and negative values indicate deposition and erosion, respectively. *τ*_*c*_ and *τ*_*w*_ are the average values of daily BSS at each station (e.g. Fig. 2a and b). The magnitude of daily bed-level dynamics reflects the mean level of absolute bed-level changes, and the error bars indicate the standard deviation of this magnitude due to temporal variation. D50 was measured by monthly by collecting and analysing surface sediment (top 2 cm) at each station. The error bars indicate the standard deviation of D50.

When look into the spatial distribution of relative bed-level positions, an overall steepening trend at both sites can be observed, i.e. erosion at the lower stations and deposition at the upper stations (Fig. 3a and b). This morphodynamic trend may be attributed to the *τ*_*c*_ distribution, which is highest at the lowest stations and decreases gradually towards the upper stations at both sites.

The magnitude of *τ*_*w*_ is contrastingly different at these two sites due to the contrasting wave exposure. At the sheltered Baarland site, *τ*_*w*_ is close to zero at all stations. At the wave-exposed Zuigdors site, *τ*_*w*_ is much higher and has a unimodal distribution over the tidal flat, with its peak at station Z6. *τ*_*w*_ exceeds *τ*_*c*_ at the upper stations (i.e. Z1-Z6, 1.8 m NAP to 1.0 m NAP), but it should be noted that *τ*_*c*_ remains the main hydrodynamic forcing at this site, as for the most part of the intertidal area (i.e., Z7 to Z9; 1.0 m NAP to ca. −2 m NAP) *τ*_*c*_ is stronger than *τ*_*w*_ (Fig. 3a). Additionally, the spatial steepening trend of mean bed-level position dynamics is likely controlled by *τ*_*c*_ not by *τ*_*w*_ (Fig. 3a and b).

With different hydrodynamic forcing, daily bed-level dynamics at both sites has comparable levels and similar seaward increasing patterns (Fig. 3c and d). The mean magnitude of daily bed-level dynamics is in the range of 0.1–1.3 cm, indicating active short-term bed-level fluctuations at both sites. For stations Z2-Z6 and B2, the overall bed-level dynamics indicated by the mean bed-level positions is small, i.e. within 3 cm, but their mean daily bed-level dynamics magnitude exceeds 0.25 cm, suggesting frequently alternating erosion and deposition. The mean magnitude of daily bed-level dynamics and its variations (i.e. error bars in Fig. 3c and d) generally increase towards the seaward direction. It is noticed that both *τ*_*c*_ and the mean magnitude of daily bed-level dynamics generally increase towards the sea. However, the correlations between these two are insignificant for both site, with r = 0.884, p = 0.108 > 0.10, n = 9 for the Zuidgors site and r = 0.743, p = 0.467 > 0.10, n = 3 for the Baarland site.

The monthly surveyed median bed sediment grain size (D50) shows a seaward increase pattern as well. Notably, D50 at the Zuigors site is general larger than that at the Baarland site. Especially, the two most seaward stations at the Zuidgors stations have much coarser surface sediment than other stations. The difference in D50 at these two sites may be caused by the stronger hydrodynamic forces at the Zuidgors site. Based on the obtained grain size data, the critical BSS for initiation of sediment motion (*τ*_*cri*_) is in the range of 0.09–0.21 Pa for the Zuigors site, and around 0.09 Pa for the Baarland site, respectively. The difference in *τ*_*cri*_ may explain why the sheltered Baarland site had comparable daily bed-level activity as the exposed Zuidgors site, even though its BSS is clearly lower (Fig. 3a and b). Thus, even though the Baarland site experiences smaller forcing, the bed sediment can be more easily suspended and transported due to the smaller *τ*_*cri*_, leading to similar level of short-term bed-level dynamics.

### Correlation between BSS spatial distribution and spatial morphodynamic patterns

Correlation analysis is conducted to reveal the relation between hydrodynamic forcing (wave and tide current) distribution and spatial morphodynamic patterns. The spatial morphodynamic patterns can be denoted by (*1*) long-term morphological variations indicated by the spatial distribution of mean relative bed positions, and (*2*) short-term morphodynamics indicated by the spatial distribution of daily bed-level dynamics magnitude. Prior to the correlation analysis, spatial autocorrelation level of the original data sets has been examined to ensure independent data for the statistics analysis. When necessary, the original data sets were subsampled prior to the correlation analysis.

**Figure 4:**
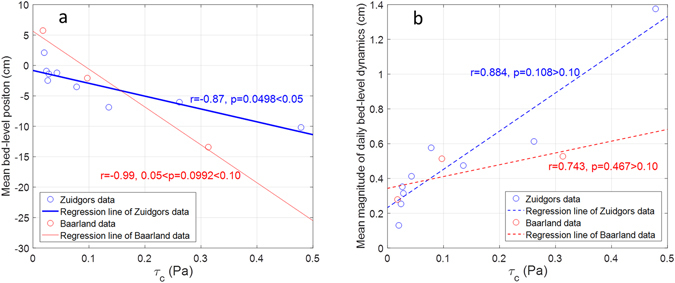
Correlation between *τ*_*c*_ distribution and mean relative bed positon (plane **a**), and correlation between *τ*_*c*_ and the mean magnitude of daily bed-level dynamics (plane **b**) at both sites. The data of the *τ*_*c*_ distribution, mean relative bed positons and the mean magnitude of daily bed-level dynamics is shown in the Fig. 3. The significant and marginally significant correlation is shown as thick solid line and thin solid line, respectively. The insignificant correlations are shown as dashed lines.

We show that the correlation coefficients (*r*) between *τ*_*c*_ distribution and the mean relative bed positions is −0.87 (*p* = 0.0498 < 0.05, *n* = 5, subsampled) and −0.99 (0.05 < *p* = 0.0992 < 0.10, *n* = 3) at the Zuidgors and Baarland sites, respectively (Fig. 4a). Thus, the correlation is significant and marginally significant (i.e. 0.05 ≤ *p* < 0.10) at these two site. These strong correlations imply the regulating role of *τ*_*c*_ on the relative long-term bed-level dynamics. However, the correlation between *τ*_*c*_ distribution and the distribution of daily bed dynamics magnitude is insignificant at the Zuidgors (*r* = 0.884, *p* = 0.108 > 0.10, *n* = 5, subsampled) and the Baarland and (*r* = 0.743, *p* = 0.467 > 0.10, *n* = 3) site, respectively (Fig. 4b). Hence, the impact of *τ*_*c*_ distribution on the short-term bed-level dynamics pattern is unimportant.

As the *τ*_*w*_ is almost negligible at the sheltered Baarland site (Fig. 3b), the correlation analysis between *τ*_*w*_ distribution and spatial morphodynamic patterns are excluded for this site. For the exposed Zuidgors site with larger *τ*_*w*_ (e.g. Fig. 3a), the correlation coefficient between the *τ*_*w*_ distribution and the distribution of mean relative bed positions is *r* = −0.1295 (*p* = 0.8671 > 0.10, *n* = 5, subsampled). The correlation coefficient between the *τ*_*c*_ distribution and the distribution of daily bed-level dynamics magnitude is *r* = 0.2619 (*p* = 0.6572 > 0.10, *n* = 5, subsampled). These two insignificant correlations show that the spatial distribution of *τ*_*w*_ does not exerts a strong impact on the spatial morphodynamic patterns.

### Correlation between local tidal/wave forcing and daily bed-level dynamics

The correlation analysis between the local hydrodynamic forcing and daily bed-level dynamics reveals the important impact of local wave forcing at the stations of the exposed site (expect for Z4 and Z9) (Table 1). The local daily wave and tidal forcing were represented by the 90th percentile value of the daily *τ*_*w*_ and *τ*_*c*_, respectively. These values have been identified as a useful value account for both force magnitude and fraction of time that force is large^15,17^.

**Table 1:** The correlation between daily bed dynamics and wave/tidal forcing at each station.

Stations	Z1	Z2	Z3	Z4	Z5	Z6	Z7	Z8	Z9	B1	B2	B3
*r* between daily bed dynamics and ***τ***_*w*_	−***0***.***12***	−***0***.***13***	−***0***.***16***	−0.07	−***0***.***22***	−***0***.***19***	−***0***.***27***	−***0***.***19***	−0.04	−0.08	−0.01	0.00
*p*-value	***0***.***03***	***0***.***01***	***0***	0.16	***0***	***0***	***0***	***0***	0.37	0.11	0.88	0.93
*r* between daily bed and ***τ***_*c*_	−0.02	−0.09	−0.03	−***0***.***11***	−0.03	0	0.01	−0.02	0.01	−0.01	−0.02	−0.04
*p*-value	0.76	0.05	0.52	***0***.***02***	0.59	0.96	0.85	0.71	0.84	0.83	0.74	0.48

Significant correlations are indicated by bold and italic fonts to enable easy visual detection.

For most of the stations at Zuidgors (expect for Z4 and Z9), the daily bed dynamics is significantly correlated to the local wave forcing, whereas the correlation between the daily bed dynamics and the local tidal forcing is insignificant (expect for Z4), implying that daily bed-level dynamics is not linked to local tidal forcing. The correlations between *τ*_*w*_ and daily bed-level dynamics at Zuidgors stations are all negative. Thus, larger *τ*_*w*_ is related to erosion while smaller *τ*_*w*_ is associated with deposition. For the Baarland stations, the correlations between daily bed-level dynamics and daily tidal or wave forcing are all insignificant.

### BSS and bed-level dynamics during storm events

**Figure 5:**
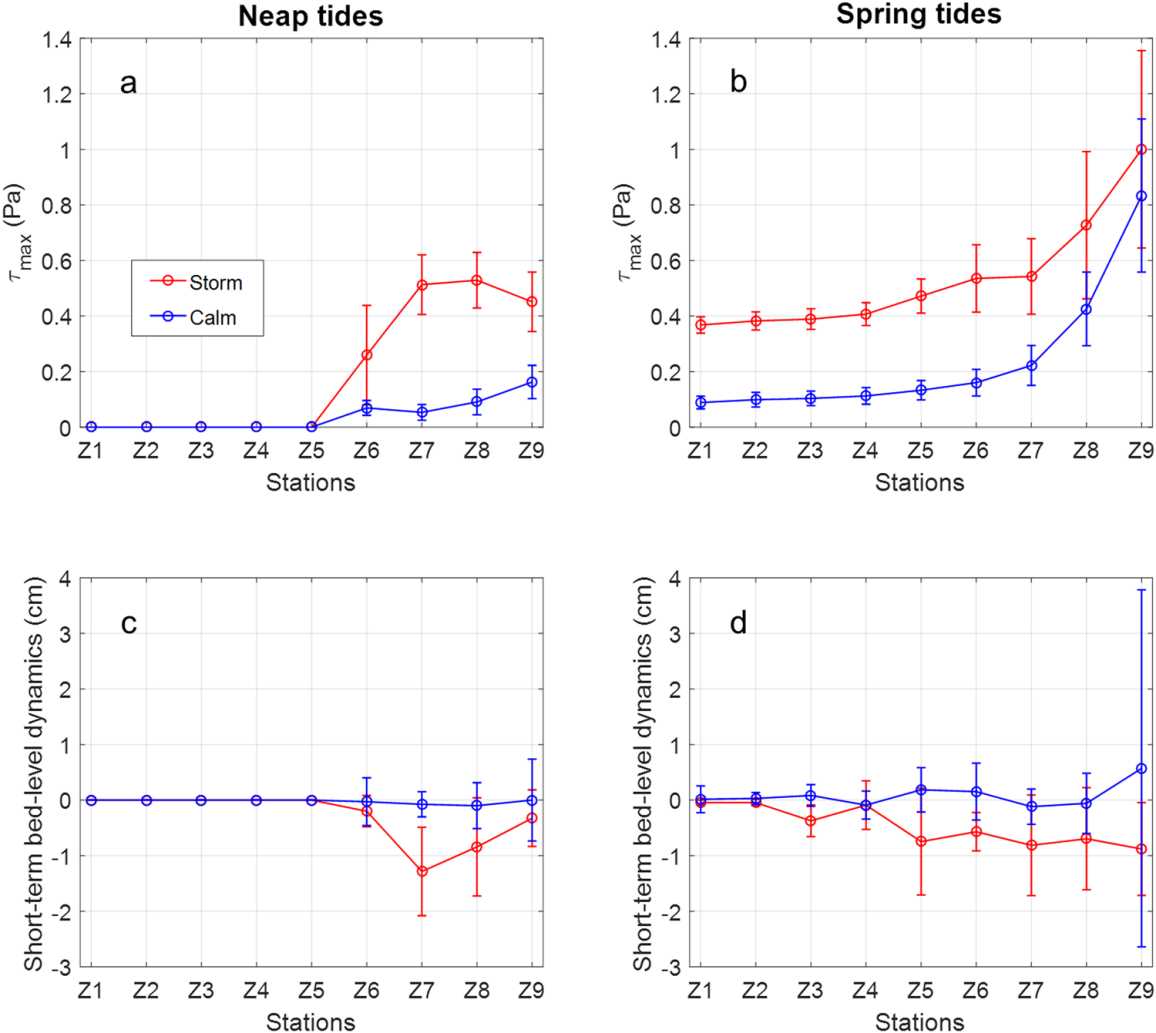
Comparison of the total BSS (*τ*_max_) (panel a and b), and the corresponding short-term bed-level dynamic (panel c and d) during storm and calm conditions over neap or spring tides at the exposed Zuidgors site. The positive and negative values in bed-level dynamics indicate deposition and erosion, respectively. The plotted data is the mean value over a few tidal cycles in the same category (e.g. neap-storm, neap-calm and etc.), and the error bars are the corresponding standard deviations. The total number of neap-storm, neap-calm, spring-storm and spring-calm tidal cycles is 4, 18, 3 and 29, respectively. Storm condition is defined as when the incident significant wave height at Z9 exceeds 0.5 m, whereas the calm condition is defined as when the incident significant wave height is below 0.1 m.

The Zuidgors site is directly exposed to waves, where storm events can lead to sudden increase of the total BSS (*τ*_max_) and bed erosion (Fig. 5). The impact of storms on *τ*_max_ and bed-level dynamics is clearly regulated by the vertical tide levels. When storms occur during neap tide, *τ*_max_ increases from station Z9 towards station Z8 and Z7, and then it drops rapidly towards station Z5, where the water is very shallow (Fig. 5a). From station Z5 upwards, *τ*_max_ reduces to zero because of the limited neap high tide level. The *τ*_*c*_ peaks around stations Z7–Z8 may be attributed to the wave breaking processes. During neap-calm tidal cycles, *τ*_max_ is much smaller than that of neap-storm conditions, and it reduces almost monotonously from station Z9 to Z5. As a consequence, the bed erosion during neap-storm condition is much more apparent compared to neap-calm condition, especially at the Z7 station with high *τ*_max_ (Fig. 5c).

In spring-storm conditions, *τ*_max_ is affected across the whole tidal flat (Fig. 5c). Due to the increased incident waves, *τ*_max_ during spring-storm conditions is also stronger comparing to the spring-clam conditions, but they share a similar monotonically decreasing pattern from station Z9 to Z1. During spring-storm tidal cycles, increased erosion was spread out to almost all stations compared to the spring-calm conditions (Fig. 5d). The general erosion pattern across the tidal flat is also different from the localized erosion pattern in neap-storm conditions.

Although the storm events have a strong impact on bed-level per event, it should be noted that over the 15 months monitoring period, the number of stormy tidal cycles (i.e. incident wave height >0.5 m, 17 tidal cycles) is much smaller than the number of calm tidal cycles (i.e. incident wave height <0.1 m, 292 tidal cycles).

## Discussion

Based on high spatiotemporal resolution data, we have extended on previous studies and reveal detailed pattern and hydrodynamic drivers of bed-level dynamics on two tidal flats with contrasting wave exposure. We show that the magnitude of short-term bed-level variations is high comparing to the net bed-level changes at both study sites. It implies that the intertidal environment has a high level of bed-level dynamics for the establishment of new vegetation or benthos^23,24,26–29^. It is apparent that the magnitude of daily bed-level dynamics increases towards the seaward direction with the enhanced total BSS from tidal current and wave (*τ*_max_). Thus, the chance of vegetation or benthos establishment decreases if they settle at lower elevations with greater bed dynamics and longer inundation time^25,28^. This finding confirms previous schematisation of short-term sediment dynamics over tidal flats, which causes lateral salt marsh migrations^29^. It may also partly explain why the newly established saltmarsh areas are generally found in areas above a certain elevation threshold^35^.

The relative importance of wave and tidal forcing on tidal flat morphodynamics is often discussed in systems with different tidal ranges^15,16,30,36–38^. Tidal force is the dominating force regardless of wave exposure conditions. The tide forcing regulates the overall morphodynamic pattern (indicated by the distribution of mean bed-level positions), as their correlation between tide forcing distribution and the overall morphodynamic pattern is significant or marginally significant at these two sites. The overall tidal flat morphodynamic trend has a steepening trend. It confirms previous findings that tide dominance can lead to net landward sediment transport and deposition in the upper tidal flat^15–17^. Additionally, seasonal variations in the tidal forcing can be observed in the current study, which is similar to the previous studies in meso and marco-tidal environment^39,40^.

Although the general morphodynamic pattern over the whole monitoring period is set by *τ*_*c*_, the daily bed-level fluctuation at most of the exposed stations is significantly related to the local wave forcing variation (Table 1). Thus, during the process of mean bed-level variation (regulated by *τ*_*c*_), the oscillating wave forcing induces high-frequency bed-level fluctuations. This results also support our hypothesis that the strong wave forcing at the exposed site exerts a significant impact on the short-term bed-level dynamics, while the relative weak wave forcing at the shelter site does not have an apparent influence on short-term bed-level changes. The effect of wave forcing is most apparent during storm events at the exposed site. The total forcing (*τ*_max_) is suddenly enhanced during storm events, which leads to abrupt bed erosion. However, the storm events are rare and transient. Thus, they do not impose a consistent effect on the bed-level variation as tide forcing does. Furthermore, by comparing the neap-storm and spring-storm condition, we also show the storm impacts are largely regulated by the vertical tidal level.

Thus, the respective roles of tidal and wave forcing at the two contrasting sites can be summarised as: (*1*) tidal forcing sets the general morphological evolution pattern over middle to long-term at both sites; (*2*) wave forcing exerts a strong impact on short-term bed-level fluctuations at the exposed Zuidgors site, but a similar effect is not seen at the sheltered Baarland with reduced wave height; (*3*) the storm impacts at the exposed site are largely regulated by vertical tidal levels. Despite the importance of wave and tide forcing, not all the variations in daily bed-level dynamics can be explained by these two forces. For instance, the daily bed-level dynamics at the shelter Baarland site is not correlated with either tidal or wave force (Table 1), and there is no clear relation between hydrodynamic force level and bed level change magnitude between these two different sites (Fig. 2). It suggests other factors including e.g. biostabilization/bioturbation, external sediment supply as well as rain and drainage may be also at work^15,20,41–46^. Thus, further studies are needed to gain a comprehensive understanding of short-term bed-level dynamics.

Previous studies on short-term intertidal morphodynamic behaviour were generally confined by the number of monitoring stations or monitoring period^20–22^. In rare instances, high-resolution bed-level data matrixes were obtained in pervious manual surveys, which have revealed interesting intertidal morphodynamic responses to various hydrodynamic conditions^34,47^. In the current study, sufficient spatial (12 stations) and temporal (nearly 15 months) monitoring coverage is achieved by applying the new automatic SED-sensors^33^. Daily bed-level dynamics and BSS distribution are obtained by combining the observation with hydrodynamic modelling, but the required labour is substantially reduced. This study may set a template for future field studies on short-term bed-level dynamics with high spatiotemporal resolution. It is noted that without a proper method or tool, in-depth space-time analysis is difficult. Thus, the obtained unique dataset invites detailed morphodynamic modelling studies to improve our understanding and predicative ability of short-term bed-level fluctuations.

A better understanding of short-term intertidal bed-level dynamics is important for the study of long-term morphological evolution and bio-morphological processes on tidal flats^15,29,30^. Based on high-resolution observation and validated hydrodynamic models, we demonstrate the seaward increasing pattern of daily bed-level dynamics and hydrodynamic forcing at both field sites. The regulating role of tidal forcing on long-term intertidal morphology, as well as the important impact of wave forcing on short-term bed-level fluctuations at the wave-exposed site are highlighted. Storm-induced BSS surge and aggravated erosion are also systematically demonstrated. Our findings provide a spatially explicit understanding of sediment dynamics on tidal flats with contrasting wave exposure, which are valuable in interpreting the key ecological processes such as seedling establishment and cliff invitation.

## Study area

Current study was conducted on tidal flat Zuidgors and Baarland (Fig. 1a). These two tidal flats are located in the north bank of the Westerschelde Estuary, near the town of Ellewoutsdijk, in the south-west of the Netherlands. The Westerschelde is a meso to macrotidal estuary, and the maximum depth-averaged current velocities in main channels are typically 1–1.5 m/s^48^. At both sites, the mean tide range is ca. 4.1 m and the mean high water level (MHWL) 2.3 m NAP (Normal Amsterdam Peil). Both sites were composed of marginal saltmarsh areas and unvegetated tidal flats, which were assessable from land during low tide (Fig. 1c). Notably, Baarland is sheltered from waves by a seaward shoal (Fig. 1c), whereas Zuidgors is exposed to relatively large waves induced by prevailing south-westerly winds^30^.

## Methods

### Field measurements

In the current study, wave parameters and bed-level dynamics were measured synchronously. Nearly 15 months of measurements, from 11-Oct-2013 to 06-Jan-2015 were executed in 11 consecutive periods. Each period lasted for 25–56 days. Between two measuring periods, there was a few days gap for retrieving and reinstalling instruments. During the measurement gaps, no data was acquired. Tidal current measurements were conducted from 19-Dec-2013 to 16-Jan-2014, which covered two complete spring-neap tidal cycles.

The wave and tidal current measurements were conducted by pressure sensors (OSSI-010–003C, Ocean Sensor Systems, Inc.) and ADCPs (Acoustic Doppler Current Profiler), respectively. Bed-level changes were monitored automatically by SED-sensors, which were developed by Royal Netherlands Institute for Sea Research (NIOZ)^33^. The measuring window of the SED-sensor is daytime during low-tide. Values of bed level were therefore obtained for each day. For some of the monitoring period, SED-sensor failure led to no daily bed-level data (Fig. 2). Monthly-obtained differential GPS data was used as substitution to record bed-level changes, and the data was interpolated to maintain the daily resolution for further analysis.

Surface sediment samples (upper 2 cm) were taken monthly from Jan. 2014 to Jan. 2015, except Sep. and Dec. 2014. The median grain size (D50) of these sediment samples was analysed in the laboratory of Royal Netherlands Institute for Sea Research (NIOZ) using a laser particle sizer (Malvern Master Sizer 2000). The critical BSS (*τ*_*cri*_) that initiates sediment motion was determined for each station based on these sediment samples data following the method described in van Rijn^49^. The effects of cohesiveness and packing are considered. For the Zuigors site, the proportion of clay fraction (<8 μm) is estimated to be 0.1 for the stations with D50 ≥ 62 μm. As for the Baarland site, D50 at all stations was ≤62 μm. Thus, *τ*_*cri*_ can be obtained directly.

### Hydrodynamic modelling and BSS quantification

The cross-shore and long-shore current modelling was conducted following the method described in Le Hir^36^. Wave modelling was carried out by using SWAN (Simulating WAves Nearshore)^50^. The set-up and validation of these hydrodynamic models are included in the Supplementary Information. Both models are generally in good agreement with the measured wave or velocity data (see Supplementary Figs S2 and S3). The modelling errors in the velocity modelling may be attributed to the factors (e.g. wind or wave driven velocity) that are not included in the current simple model (Supplementary Fig. S3). These validated models were used to quantify *τ*_*c*_, *τ*_*w*_ and *τ*_max_. The maximum BSS due to the combined effect of wave and tidal current (*τ*_max_) was obtained following Soulsby’s method^51^.

### Data a**s**sessment

The relation between hydrodynamic forcing and bed-level dynamics was primarily investigated by correlation analysis. As any statistical analysis requires independent data source, spatial autocorrelation analysis was performed prior to the correlation analysis to evaluate the autocorrelation levels of the original BSS and bed-level data sets. The spatial autocorrelation level was measured by Moran’s I^52^, which is a function of distance. When the autocorrelation level of an original data set exceeded the 1/e threshold, it was subsampled until its autocorrelation level drops below this threshold. The results of the spatial autocorrelation levels and the subsequent subsampling procedure are detailed in the Supplementary Information. In all correlation analyses, we distinguish 3 significant levels: significant when *p* < 0.05, marginally significant when 0.05 ≤ *p* < 0.10, insignificant 0.10 ≤ *p*^53^.

## Electronic supplementary material

Supplementary Information

## References

[1] Allen JRL (2000). Morphodynamics of Holocene salt marshes: A review sketch from the Atlantic and Southern North Sea coasts of Europe. Quat. Sci. Rev..

[2] Friess DA (2016). Mangrove forests. Curr. Biol..

[3] Zhu Z (2016). Sprouting as a gardening strategy to obtain superior supplementary food: Evidence from a seed-caching marine worm. Ecology.

[4] Temmerman S (2013). Ecosystem-based coastal defence in the face of global change. Nature.

[5] Bouma TJ (2014). Identifying knowledge gaps hampering application of intertidal habitats in coastal protection: Opportunities & steps to take. Coast. Eng..

[6] Möller I (2014). Wave attenuation over coastal salt marshes under storm surge conditions. Nat. Geosci..

[7] Van Eerden MR, Drent RH, Stahl J, Bakker JP (2005). Connecting seas: western Palaearctic continental flyway for water birds in the perspective of changing land use and climate. Glob. Change Biol..

[8] Laursen K, Asferg KS, Frikke J, Sunde P (2009). Mussel fishery affects diet and reduces body condition of Eiders Somateria mollissima in the Wadden Sea. J. Sea Res..

[9] Laursen, K. *et al*. *Migratory birds*. 1–18 (2009).

[10] Kirwan M, Temmerman S (2009). Coastal marsh response to historical and future sea-level acceleration. Quat. Sci. Rev..

[11] Kirwan ML, Guntenspergen GR (2010). Influence of tidal range on the stability of coastal marshland. J. Geophys. Res. Earth Surf..

[12] Mariotti, G. & Fagherazzi, S. A numerical model for the coupled long-term evolution of salt marshes and tidal flats. *J*. *Geophys*. *Res*. *F Earth Surf*. **115** (2010).

[13] Fagherazzi, S. *et al*. Numerical models of salt marsh evolution: Ecological, geomorphic, and climatic factors. *Rev*. *Geophys*. **50** (2012).

[14] Schuerch M, Vafeidis A, Slawig T, Temmerman S (2013). Modeling the influence of changing storm patterns on the ability of a salt marsh to keep pace with sea level rise. J. Geophys. Res. F Earth Surf..

[15] Friedrichs, C. T. Tidal Flat Morphodynamics: A Synthesis. In *Treatise on Estuarine and Coastal Science* (eds Wolanski, E. & McLusky, D.) 137–170 (Academic Press, 2011).

[16] Green MO, Coco G (2014). Review of wave-driven sediment resuspension and transport in estuaries. Rev. Geophys..

[17] Hu Z, Wang ZB, Zitman TJ, Stive MJF, Bouma TJ (2015). Predicting long-term and short-term tidal flat morphodynamics using a dynamic equilibrium theory. J. Geophys. Res.-Earth Surf..

[18] Hunt S, Bryan KR, Mullarney JC (2015). The influence of wind and waves on the existence of stable intertidal morphology in meso-tidal estuaries. Geomorphology.

[19] Maan DC, Van P, Wang ZB, De V (2015). Do intertidal flats ever reach equilibrium?. J. Geophys. Res. F Earth Surf..

[20] Whitehouse RJS, Mitchener HJ (1998). Observations of the morphodynamic behaviour of an intertidal mudflat at different timescales. Geol. Soc. Lond. Spec. Publ..

[21] Zhu Q, Yang S, Ma Y (2014). Intra-tidal sedimentary processes associated with combined wave–current action on an exposed, erosional mudflat, southeastern Yangtze River Delta, China. Mar. Geol..

[22] Shi BW, Yang SL, Wang YP, Yu Q, Li ML (2014). Intratidal erosion and deposition rates inferred from field observations of hydrodynamic and sedimentary processes: A case study of a mudflat-saltmarsh transition at the Yangtze delta front. Cont. Shelf Res..

[23] Bouma H, Duiker JMC, De Vries PP, Herman PMJ, Wolff WJ (2001). Spatial pattern of early recruitment of Macoma balthica (L.) and Cerastoderma edule (L.) in relation to sediment dynamics on a highly dynamic intertidal sandflat. J. Sea Res..

[24] Nambu R, Saito H, Tanaka Y, Higano J, Kuwahara H (2012). Wave actions and topography determine the small-scale spatial distribution of newly settled Asari clams Ruditapes philippinarum on a tidal flat. Estuar. Coast. Shelf Sci..

[25] Balke T (2013). Seedling establishment in a dynamic sedimentary environment: A conceptual framework using mangroves. J. Appl. Ecol..

[26] Balke T (2013). Cross-shore gradients of physical disturbance in mangroves: Implications for seedling establishment. Biogeosciences.

[27] Balke T, Herman PMJ, Bouma TJ (2014). Critical transitions in disturbance-driven ecosystems: Identifying windows of opportunity for recovery. J. Ecol..

[28] Hu Z (2015). Windows of opportunity for salt marsh vegetation establishment on bare tidal flats: The importance of temporal and spatial variability in hydrodynamic forcing. J. Geophys. Res. G Biogeosciences.

[29] Bouma TJ (2016). Short-term mudflat dynamics drive long-term cyclic salt marsh dynamics. Limnol. Oceanogr..

[30] Callaghan DP (2010). Hydrodynamic forcing on salt-marsh development: Distinguishing the relative importance of waves and tidal flows. Estuar. Coast. Shelf Sci..

[31] Hunt S, Bryan KR, Mullarney JC, Pritchard M (2016). Observations of asymmetry in contrasting wave- and tidally-dominated environments within a mesotidal basin: implications for estuarine morphological evolution. Earth Surf. Process. Landf..

[32] Andersen TJ, Pejrup M, Nielsen AA (2006). Long-term and high-resolution measurements of bed level changes in a temperate, microtidal coastal lagoon. Mar. Geol..

[33] Hu Z, Lenting W, van der Wal D, Bouma TJ (2015). Continuous monitoring bed-level dynamics on an intertidal flat: Introducing novel, stand-alone high-resolution SED-sensors. Geomorphology.

[34] Yang SL (2003). Morphological Response of Tidal Marshes, Flats and Channels of the Outer Yangtze River Mouth to a Major Storm. Estuaries.

[35] Wang, C. & Temmerman, S. Does bio-geomorphic feedback lead to abrupt shifts between alternative landscape states? An empirical study on intertidal flats and marshes. *J*. *Geophys*. *Res*. *Earth Surf*. n/a–n/a, 10.1002/jgrf.20027 (2013).

[36] Le Hir P (2000). Characterization of intertidal flat hydrodynamics. Cont. Shelf Res..

[37] D’Alpaos A, Carniello L, Rinaldo A (2013). Statistical mechanics of wind wave-induced erosion in shallow tidal basins: Inferences from the Venice Lagoon. Geophys. Res. Lett..

[38] Zhu Q, van Prooijen BC, Wang ZB, Ma YX, Yang SL (2016). Bed shear stress estimation on an open intertidal flat using *in situ* measurements. Estuar. Coast. Shelf Sci..

[39] Allen JRL, Duffy MJ (1998). Medium-term sedimentation on high intertidal mudflats and salt marshes in the Severn Estuary, SW Britain: The role of wind and tide. Mar. Geol..

[40] Harrison SR, Bryan KR, Mullarney JC (2017). Observations of morphological change at an ebb-tidal delta. Mar. Geol..

[41] Herman PMJ, Middelburg JJ, Heip CHR (2001). Benthic community structure and sediment processes on an intertidal flat: Results from the ECOFLAT project. Cont. Shelf Res..

[42] Le Hir P, Monbet Y, Orvain F (2007). Sediment erodability in sediment transport modelling: Can we account for biota effects?. Cont. Shelf Res..

[43] Dai Z, Liu JT (2013). Impacts of large dams on downstream fluvial sedimentation: An example of the Three Gorges Dam (TGD) on the Changjiang (Yangtze River). J. Hydrol..

[44] Dai Z, Liu JT, Wei W, Chen J (2014). Detection of the Three Gorges Dam influence on the Changjiang (Yangtze River) submerged delta. Sci. Rep..

[45] Dai Z, Fagherazzi S, Mei X, Gao J (2016). Decline in suspended sediment concentration delivered by the Changjiang (Yangtze) River into the East China Sea between 1956 and 2013. Geomorphology.

[46] Green MO, Coco G (2007). Sediment transport on an estuarine intertidal flat: Measurements and conceptual model of waves, rainfall and exchanges with a tidal creek. Estuar. Coast. Shelf Sci..

[47] Fan D, Guo Y, Wang P, Shi JZ (2006). Cross-shore variations in morphodynamic processes of an open-coast mudflat in the Changjiang Delta, China: With an emphasis on storm impacts. Cont. Shelf Res..

[48] Wang ZB, Jeuken MCJL, Gerritsen H, de Vriend HJ, Kornman BA (2002). Morphology and asymmetry of the vertical tide in the Westerschelde estuary. Cont. Shelf Res..

[49] van Rijn LC (2007). Unified view of sediment transport by currents and waves. I: Initiation of motion, bed roughness, and bed-load transport. J. Hydraul. Eng..

[50] Booij N, Ris RC, Holthuijsen LH (1999). A third-generation wave model for coastal regions 1. Model description and validation. J. Geophys. Res. C Oceans.

[51] Soulsby, R. Dynamics of marine sands: a manual for practical applications. (Telford, 1997).

[52] Fortin, M.-J. & Dale, M. R. T. *Spatial Analysis*: *A Guide for Ecologists*. (Cambridge University Press, 2005).

[53] La Nafie YA, de los Santos CB, Brun FG, van Katwijk MM, Bouma TJ (2012). Waves and high nutrient loads jointly decrease survival and separately affect morphological and biomechanical properties in the seagrass Zostera noltii. Limnol. Oceanogr..

