# Publisher Correction: Patterns and drivers of daily bed-level dynamics on two tidal flats with contrasting wave exposure

**DOI:** 10.1038/s41598-017-18749-1

**Published:** 2018-01-08

**Authors:** Zhan Hu, Peng Yao, Daphne van der Wal, Tjeerd J. Bouma

**Affiliations:** 1 Institute of Estuarine and Coastal Research, School of Marine Science, Sun Yat-sen University, Guangzhou, 510275 China; 2 Guangdong Provincial Key Laboratory of Marine Resources and Coastal Engineering, Guangzhou, 510275 China; 3 State-province Joint Engineering Laboratory of Estuarine Hydraulic Technology, Guangzhou, 510275 China; 4 Royal Netherlands Institute for Sea Research (NIOZ), P.O. Box 140, 4400 AC Yerseke, Netherlands

Correction to: *Scientific Reports* 10.1038/s41598-017-07515-y, published online 02 August 2017

The PDF version of this Article was inadvertently replaced after publication. This has now been corrected; the HTML version of the paper was correct from the time of publication.

