# Supplementary material for: Patterns and drivers of daily bed-level dynamics on two tidal flats with contrasting wave exposure
Source: Sci Rep. 2017 Aug 2;7:7088. doi: 10.1038/s41598-017-07515-y (PMC5541070; doi:10.1038/s41598-017-07515-y)
Supplement: Supplementary file 1 — Supplementary Information [file 41598_2017_7515_MOESM1_ESM.pdf]

Supplementary Information

Patterns and drivers of daily bed-level dynamics on two tidal flats with contrasting wave exposure

Zhan Hu[1,2,3,*,+], Peng Yao[1,2,3,+], Daphne van der Wal[4], Tjeerd J. Bouma[4]

[1]Institute of Estuarine and Coastal Research, School of Marine Science, Sun Yat-sen University, Guangzhou 510275, China
[2]Guangdong Provincial Key Laboratory of Marine Resources and Coastal Engineering, Guangzhou 510275, China
[3]State-province Joint Engineering Laboratory of Estuarine Hydraulic Technology, Guangzhou 510275, China
[4]Royal Netherlands Institute for Sea Research (NIOZ), P.O. Box 140, 4400 AC Yerseke, Netherlands
*Corresponding author: huzh9@mail.sysu.edu.cn
[+]These authors contributed equally to this work

This Supporting Information includes ***bed-level data sets at all stations***, ***spatial autocorrelation check*** and ***hydrodynamic model setup and validation.***

Bed-level data sets at all stations

The bed-level monitoring data obtained by the SED-sensors[1] at all 12 stations is demonstrated in the Fig. S1. During SED-sensor failure, monthly-obtained differential GPS data was used as substitution to record bed-level dynamics.

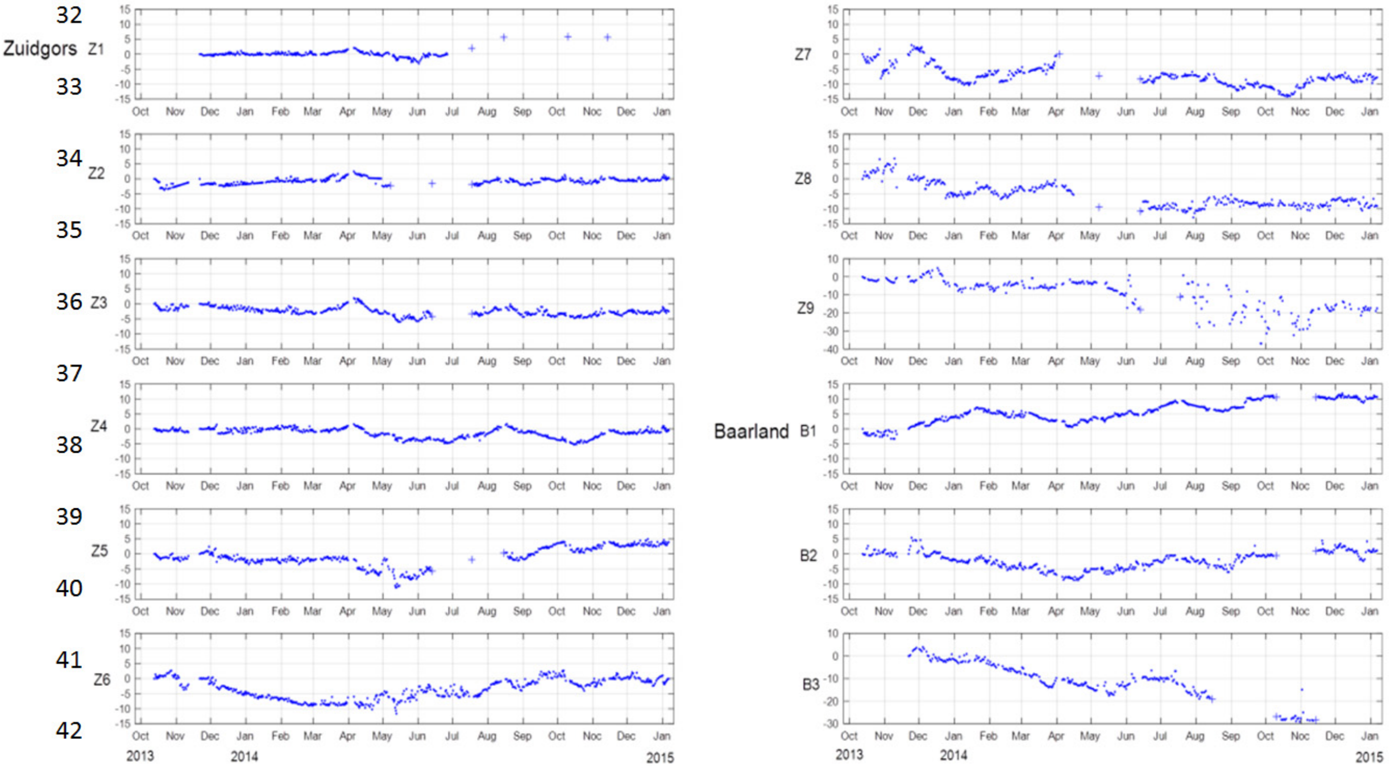

Figure S1.Bed-level positions (in cm) observed at 12 stations at the Zuidgors and Baarland sites by SED-sensors[1]. The initial bed-level position is at '0' at each station. The '+' sign indicates the differential GPS data, which was used as substitution to the SED-sensor data.

Spatial autocorrelation check

Based on the EOF analysis results, we conducted correlation analysis of the spatial eigenfunctions of bed level dynamics and BSS to show the linkage between these two. Like any statistical analysis, the correlation analysis requires independent data sets to avoid inflated statistical significance. Thus, prior to the correlation analysis, we evaluated the spatial autocorrelation level of the obtained spatial eigenfunctions using Moran 's I[2]. If an original data set has a Moran 's I higher than 1/e or lower than -1/e, it needs to be subsampled to obtain an independent data set with a lower spatial autocorrelation level. Moran 's I (i.e. $I(d)$) is a function

of distance (d) between monitoring station sequence[2]:

$$I(d)=\frac{\frac{1}{W}\sum_{i=1}^{n}\sum_{j=1}^{n}W_{ij}(x_i-\bar{x})(x_j-\bar{x})}{\frac{1}{n}\sum_{i=1}^{n}(x_i-\bar{x})^2} \qquad \text{(S1)}$$

where x_i and x_j are the spatial eigenfunction values at station i and j, respectively; $\bar{x}$ is the spatial mean value of all the n stations; $W_{ij}=1$, when the distance between x_i and x_j is d; $W_{ij}=0$, for all other cases; W is the sum of the W_{ij} matrix. If d=1, the examined data set is the original data set. If d=2, the examined data set is the data from every other stations of the original data set. Based on this equation, the spatial autocorrelation level of the spatial eigenfunctions of τ_{max} and bed level dynamics is obtained (Table S1).

Table S1. Moran 's I values of the τ_{max} and bed level dynamics at both sites.

Item	Zuidgors		Baarland
Distance	1	2	1
τ_c	0.53	0.19	-0.07
τ_w	0.58	-0.07	-0.10
τ_{max}	0.50	0.19	-0.07
Mean bed-level position	0.50	0.33	-0.01
Mean magnitude of daily bed dynamics	0.28	0.11	-0.21

At the Baarland site, the absolute values of Moran 's I are smaller than the 1/e threshold. It indicates the original data sets (with distance equal to 1) have a low level of autocorrelation[2]. Thus, these spatial eigenfunctions can be regarded as independent data sets ready for correlation analysis. For the Zuidgors site, however, the Moran 's I values of τ_c, τ_w, τ_{max}, mean bed-level position and the magnitude of daily bed-level dynamics exceeds the 1/e threshold. Therefore, these data sets are autocorrelated, which needs subsampling for independent data. Table S1

further shows that when the sampling distance increased to 2, the Moran 's I values drop below the 1/e threshold. Therefore, we used a subsampling distance of 2 to obtain independent data sets of the spatial eigenfunctions at the Zuidgors site.

Hydrodynamic model setup and validation

Wave modelling

Hydrodynamic models were built to provide complete data sets of wave and tidal current when there was no measurement. Wind wave propagation was simulated using a spectral model, i.e. SWAN (Simulating Waves Nearshore)[3]. Wave shoaling, breaking, and bottom friction processes on tidal flats were explicitly accounted for in this model. At each site, a 1-D modelling domain was built along the measuring transect (Fig. 1). The spatial resolution of a computation domain is 1 m. Each model domain was forced by incident waves with a Joint North Sea Wave Project spectrum at the seaward boundary, while the wind-induced waves growth was excluded[4,5]. The bulk parameters of the incident waves, i.e. significant wave height and peak wave period, were provided by the most seaward station at each site. In the current study, the default model parameter setting was applied. For more details, please see http://swanmodel.sourceforge.net.

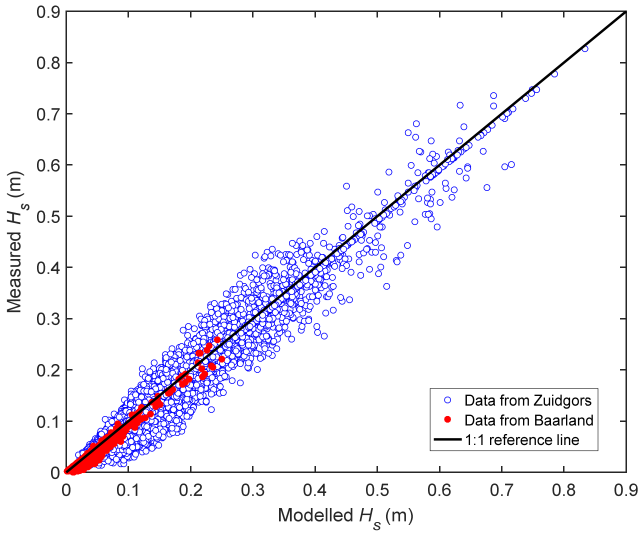

Figure S2. Comparison between the modelled and measured significant wave height (H_s)

In order to evaluate the wave model performance, the modelling results were compared to the measurements obtained in both stormy (20-Nov-2013 to 14-Jan-2014) and calm (13-Jun-2014 to 16-Jul-2014) seasons (Fig. S2). The measuring interval was 15 minutes. At both sites, the wave modelling results are in good agreement with the measurements obtained in (Fig. S3). The R^2

value and sampling size (*n*) at Zuidgors and Baarland site are 0.94 (n=19410) and 0.66 (n=8983), respectively.

Cross-shore and long-shore tidal current modelling

Both cross-shore and long-shore tidal current were modelled in the current study. Tidal current modelling shared the same computation domains of those used in wave modelling. Cross-shore current was modelled following water volume conservation, which was widely used in previous studies[6,7]. As the tide rises, the water line moves landward, resulting onshore flows. The volume of water that must pass through the vertical long-shore plane at location x must equal to the increase of water volume in the landward direction of the long-shore plane (ΔV). The water volume changes can be readily determined by tracking the tidal level fluctuation, if we assume tidal levels are always horizontal. Subsequently, the cross-shore current (u_c(x)) that cause this ΔV in a time interval of Δt can be determined as:

$$u_c(x) = \frac{\Delta V(x)}{\Delta t h(x)} \qquad \text{(S2)}$$

where $h(x)$ is the local water depth.

The long-shore tidal current at the seaward boundary (u_{l_sea}) was predicted by T_TIDE[8]. We firstly used T_TIDE to analyse the measured current velocity at the most seaward station for local tidal constituents. Then, based on the obtained tidal constituents, the T_TIDE can apply again to period u_{l_sea} at any given time. Finally, the long-shore tidal current at each location (u_l(x)) can be quantified by balancing the bed friction with the long-shore water level gradient[7]. It is further assumed that 1) the water level gradient is uniform on a tidal flat transect, since the tide propagation often has a much larger spatial scale than a typical transect; and 2) the bed friction is proportional to the square of the depth-averaged velocity. Then u_l(x) is determined as following:

$$u_l(x) = u_{l\_\mathrm{out}} \sqrt{\frac{h(\mathrm{x})}{h_{out}}} \qquad \text{(S3)}$$

where h_{out} is the water depth at the seaward boundary. The total velocity can be determined as:

$$u = \sqrt{u_c^2 + u_l^2} \qquad \text{(S4)}$$

The angle between cross-shore and long-shore current is:

$$\theta = \tan^{-1}(\frac{u_l}{u_c}) \qquad (S5)$$

At both sites, the velocity modelling results are generally consistent with the measurements (Fig. S3). The measurements were conducted from 19-Dec-2013 to 16-Jan-2014 at the stations shown in Fig. 2. The measuring interval was 10 minutes. The R^2 value and sampling size (n) of total velocity at Zuidgors and Baarland site is 0.86 (n=5721) and 0.76 (n=3111), respectively.

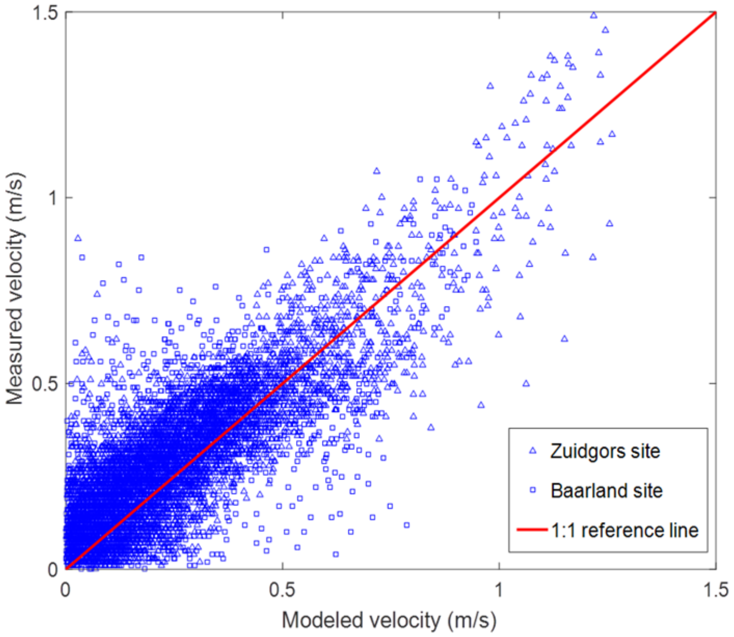

Figure S3. Comparison between the modelled and measured velocity.

Reference

1. Hu, Z., Lenting, W., van der Wal, D. & Bouma, T. J. Continuous monitoring bed-level dynamics on an intertidal flat: Introducing novel, stand-alone high-resolution SED-sensors. *Geomorphology* **245,** 223–230 (2015).

2. Fortin, M.-J. & Dale, M. R. T. *Spatial Analysis: A Guide for Ecologists*. (Cambridge University Press, 2005).

3. Booij, N., Ris, R. C. & Holthuijsen, L. H. A third-generation wave model for coastal regions 1. Model description and validation. *J. Geophys. Res. C Oceans* **104,** 7649–7666 (1999).

4. Hasselmann, K. *et al.* Measurements of wind-wave growth and swell decay during the Joint North Sea Wave Project (JONSWAP). (1973).

5. Hu, Z. *et al.* Windows of opportunity for salt marsh vegetation establishment on bare tidal flats: The importance of temporal and spatial variability in hydrodynamic forcing. *J. Geophys. Res. G Biogeosciences* **120,** 1450–1469 (2015).

6. Friedrichs, C. T. & Aubrey, D. G. in *Coastal and Estuarine Studies* (ed. Pattiaratchi, C.) **50,** 405–429 (American Geophysical Union, 1996).

7. Le Hir, P. *et al.* Characterization of intertidal flat hydrodynamics. *Cont. Shelf Res.* **20,** 1433–1459 (2000).

8. Pawlowicz, R., Beardsley, B. & Lentz, S. Classical tidal harmonic analysis including error estimates in MATLAB using T_TIDE. *Comput. Geosci.* **28,** 929–937 (2002).
